# Reasons, perceived outcomes and characteristics of second-opinion seekers: are there differences in private vs. public settings?

**DOI:** 10.1186/s12913-019-4067-4

**Published:** 2019-04-23

**Authors:** Liora Shmueli, Nadav Davidovitch, Joseph S. Pliskin, Igal Hekselman, Ran D. Balicer, Geva Greenfield

**Affiliations:** 1 Department of Health Systems Management, Ben-Gurion University of the Negev, P.O. Box 653, 84105 Beer-Sheva, Israel; 2 Department of Industrial Engineering and Management, Ben-Gurion University of the Negev, P.O. Box 653, 84105 Beer-Sheva, Israel; 3 Clalit Mushlam Health Insurance Systems, Clalit Health Services, 1 Ben Gurion, 5120149 Bnei Brak, Israel; 4 Clalit Research Institute, Clalit Health Services, 101 Arlozorov, 62098 Tel-Aviv, Israel; 5 Department of Primary Care and Public Health, School of Public Health, Imperial College London, The Reynolds Building, St. Dunstan’s Road, London, W6 8RP UK; 6 Sackler Faculty of Medicine, Tel-Aviv University, Tel-Aviv, Israel

**Keywords:** Second opinion, Private system, Public system, Utilization, Health policy, Inequalities

## Abstract

**Background:**

In most countries, patients can get a second opinion (SO) through public or private healthcare systems. There is lack of data on SO utilization in private vs. public settings. We aim to evaluate the characteristics of people seeking SOs in private vs. public settings, to evaluate their reasons for seeking a SO from a private physician and to compare the perceived outcomes of SOs given in a private system vs. a public system.

**Methods:**

A cross-sectional national telephone survey, using representative sample of the general Israeli population (*n* = 848, response rate = 62%). SO utilization was defined as seeking an additional clinical opinion from a specialist within the same specialty, on the same medical concern. We modeled SO utilization in a public system vs. a private system by patient characteristics using a multivariate logistic regression model.

**Results:**

214 of 339 respondents who obtained a SO during the study period, did so in a private practice (63.1%). The main reason for seeking a SO from a private physician rather than a physician in the public system was the assumption that private physicians are more professional (45.7%). However, respondents who obtained a private SO were neither more satisfied from the SO (*p* = 0.45), nor felt improvement in their perceived clinical outcomes after the SO (*p* = 0.37). Low self-reported income group, immigrants (immigrated to Israel after 1989) and religious people tended to seek SOs from the public system more than others.

**Conclusions:**

The main reason for seeking a SO from private physicians was the assumption that they are more professional. However, there were no differences in satisfaction from the SO nor perceived clinical improvement. As most of SOs are sought in the private system, patient misconceptions about the private market superiority may lead to ineffective resource usage and increase inequalities in access to SOs. Ways to improve public services should be considered to reduce health inequalities.

## Background

It is widely known that social inequalities in health result from factors such as socio-economic level, geographical residence, ethnicity, race end education [1, 2]. Socio-economic status, education, income and occupation are well known factors intertwined with health behaviors and health outcomes [3–5]. Differences in access to healthcare services can also explain health inequalities. Likewise, geographical residence affects access to health services [6], as most medical specialists tend to reside in central regions. The structure of the healthcare system may also introduce inequalities by access when certain services are available only to those able to purchase them privately.

These inequalities are more obvious in countries where healthcare services are mostly privatized, however they also exists in national tax-funded healthcare systems [7]. Many OECD countries have turned to a ‘public-private mix’ structure during the last decades and increased out-of-pocket co-payments, and thus increasing health inequalities [8, 9].

### Second medical opinions as a case study for inequalities in access to healthcare services

Utilization of SOs can serve as a case study for understanding inequalities in access to healthcare services. Patients may seek a SO from another physician to obtain additional information or reassurance about diagnosis, treatment or prognosis [10–12], to ascertain whether the treatment is appropriate [13–15] and also if they are dissatisfied or distrust the physician [10, 12–14, 16–22].

There are differences among countries in access, provision and payment mechanisms for SOs. In a private market such as in the US, health insurance plans may require and provide mandatory SOs for surgery. Most insurance plans will pay for at least part of the cost while Medicare will pay 80% of the cost. The Medicare health maintenance organization (HMO), which covers about 13% of the population, provides SOs in some cases before surgery with co-payment. Some plans require a referral from a primary care physician, and require seeing an in-network physician. Conversely, in countries that has a National Health Insurance (NHI) such as Canada, there is no mandatory SO requirement before surgery [23].

Although the increase of private services consumption at the expense of public services is well-studied [9], this issue was rarely examined in the context of SOs. Only few studies identified factors potentially exacerbating health disparities by identifying characteristics of patients who seek a SO, such as insurance type and coverage policy [22]. Other studies highlighted additional factors that can affect this utilization, such as living in non-central cities or suburbs of central cities or living far from an academic hospital [20, 24].

### Inequalities and access to second medical opinions in Israel

All the citizens in Israel are covered by a public NHI provided by four not-for-profit health funds, providing primary and secondary care. In parallel, more than 75% of the population are covered by voluntary, supplementary health insurance provided by the health funds or by private insurance. The proportion of private insurance and supplemental programs in Israel is significantly higher than in OECD countries (80% compared to 32.5%) [25]. Moreover, 24% (on average) of the national health expenditure in 2015 were paid directly by patients, compared to 20% in the OECD countries [26].

Patients in Israel can obtain SOs either through the private system by a private physician by paying either out-of-pocket or by receiving partial reimbursement from supplementary program offered by the health funds or by private insurance plans, or through the public system by approaching a specialist working in a community setting, through the secondary care provided by the health funds. The patients pay a quarterly co-payment for visiting a specialist. A detailed description of access to SOs in Israel appears elsewhere [27].

The boundaries between the public and private sectors are often blurred, both in relation to funding and supply. For example, in Israel the public health funds sell private supplementary health insurance [8, 9, 28] and there are cases in which the same physicians provide medical consultations in both private and public funded settings [8]. The question whether SO should be provided and covered by the government as part of the NHI becomes especially interesting in Israel, in terms of financing and provision.

Private expenditure on health for services that are not included in the public basket increased in the last decades [9], SOs have been a significant component of this rise, accounting for about 10% of the total expenditure of the supplementary health programs provided by the health funds (equivalent to $92.6 million) [29]. The increase in private expenditures and in particular for SOs, widens the gaps among social groups. These gaps in SO access can be found both in access to private vs. the public system and in the periphery vs. the center of the country [30]. We previously showed that some social groups in Israel obtain less SOs than others: immigrants, people living in peripheral areas and those who perceive their health as not so good [31].

## Methods

### Aim, design and setting of the study

We aimed to evaluate (1) the characteristics of people seeking SOs in the private system vs. the public system in Israel; (2) the reasons for seeking private SOs; and (3) the perceived outcomes of SOs given in a private system vs. a public system. Such an assessment is important to determine whether SOs are utilized in an equal, accessible and efficient way for the benefit of patients, physicians and healthcare organizations.

We conducted a cross-sectional telephone survey of the Israeli general population [32]. The survey was conducted in collaboration with the B.I. and Lucile Cohen Institute for Public Opinion Research, an academic survey unit at Tel-Aviv University, during November 2011. The interviewers followed a closed-end protocol asking participants whether they obtained a SO during the past year, their reasons for seeking a private physician vs. a physician working in public settings and the perceived outcomes from the consultation. The study was approved by the Institutional Ethics Committee for non-clinical studies (K2010/137).

### Sampling and participants

We sampled a representative random sample of the general Israeli adult population. The inclusion criterion was being 18 years old and above. The respondents were sampled by a probabilistic sampling of Israeli adult population households from layers of statistical areas, defined by socio-demographic characteristics of each area. With this way of sampling, the sample ensures representation of various population groups, particularly those with a relatively small proportion. The sample size was based on a pre-test conducted with 274 respondents, which showed that about 20% of them had obtained a SO during the last year.

We used disproportionate stratified sampling to increase the number of respondents who obtained a SO for the inferential statistics. This method allows different sampling ratios in different strata and ensures there are at least 300 respondents who obtained a SO. We over-sampled another 239 respondents who obtained a SO, using the same principles of sampling layers of statistical areas as the representative sample. Hence, the survey included a total of 848 people from the representative sample and the disproportionate stratified sampling.

We approached 984 households, out of which 609 questionnaires were completed in full (response rate 62%). 105 respondents from the representative sample (17.2% of 609) visited a physician for a SO during the study period. We calculated this rate from the representative sample only (without the disproportionate stratified sampling). With the disproportionate stratified sampling, a total of 344 respondents obtained a SO (105 from the representative sample and 239 from the oversampling). This analyses here focus on a subgroup of 339 people who obtained a SO (after excluding 5 respondents with missing data on their private-public visit place).

### Variables and measurements

The dependent, binary, variable was a self-reported setting of obtaining the SO, which was either from a physician in the public or private system. A ‘second opinion’ was defined as ‘visiting another specialist, in the same specialty, in order to get a second opinion on the same medical problem during the past 12 months’ (excluding consultations with family physicians). The covariates were socio-demographic variables: (1) gender; (2) age group; (3) educational level; (4) personal status; (5) ethnicity (Jewish/Arab); (6) religiosity (religious/secular); (7) self-reported income level; (8) socio-economic level according to residency, as defined by the Israeli Central Bureau of Statistics; (9) being an immigrant (defined as immigration to Israel after 1989); (10) country of birth; and (11) perceived health status.

### Statistical analyses

We compared the characteristics of respondents who obtained a SO in the public system to those who obtained a private SO by univariate χ2 tests. We then predicted the binary dependent variable (the setting of getting the SO: private vs. public) using a multivariate logistic regression model. To reduce the number of covariates entered into the regression we included in the null model only those covariates which were significant (*p* < 0.05) in the univariate analyses. We used backward elimination for model selection. The elimination method was Wald, based on the robust estimator of the parameter estimate covariance matrix. The threshold for elimination was 0.05. We used SPSS Version 20 for the statistical analyses.

## Results

### Characteristics of patients seeking a SO in the public system vs. the private system

Most of the respondents (214 of the 339 respondents who obtained a SO), did so in the private system (63.1%). Religious respondents sought SOs from the public system more than secular respondents. Respondents from the low self-reported income group sought more SOs from the public system relative to respondents from middle and high-income status (Table 1). Similarly, respondents objectively classified in a low socio-economic level sought SOs from the public system more than respondents from middle and high-income status. Immigrants (less than 28 years in Israel) sought SOs from the public system more than native born and established immigrants. There were no significant differences between the two groups in gender, age group, educational level, personal status, ethnicity, country of birth and perceived health status. The covariates that remained significant in the multivariate logistic regression were immigration and religiosity (Table 2). Immigrants tended to obtain SOs from the public system more than native-born and established immigrants (OR = 3.68, 95% CI 1.67–8.1), and religious people tended to obtain SOs from the public system more than secular people (OR = 0.45, 95% CI 0.27–0.76).

**Table 1:** Characteristics of respondents who sought a second opinion in the public system vs. the private system (*n* = 339)

Characteristics	Public system *n* = 125 (%)	Private system *n* = 214 (%)	*p*-value
Gender			0.892
Male	50 (37.3%)	84 (62.7%)	
Female	75 (36.6%)	130 (63.4%)	
Age group			0.29
18–39	41 (42.3%)	56 (57.7%)	
40–59	38 (31.9%)	81 (68.1%)	
60+	46 (37.4%)	77 (62.6%)	
Educational level			0.296
Basic	20 (47.6%)	22 (52.4%)	
High school	56 (36.4%)	98 (63.6%)	
Academic	49 (34.5%)	93 (65.5%)	
Missing values		1	
Personal status			0.43
Living with a partner	95 (35.4%)	173 (64.6%)	
Not Living with a partner	28 (40.6%)	41 (59.4%)	
Missing values	2		
Ethnicity			0.237
Jewish	98 (34.9%)	183 (65.1%)	
Arabic	23 (43.4%)	30 (56.6%)	
Missing values	4	1	
Religiosity			.015*
Religious	51 (45.1%)	62 (54.9%)	
Secular	70 (31.7%)	151 (68.3%)	
Missing values	4	1	
Self-reported income group			.027*
Well below the average	40 (48.2%)	43 (51.8%)	
Around the average	57 (31.5%)	124 (68.5%)	
Well above the average	8 (30.8%)	18 (69.2%)	
Missing values	20	29	
Socioeconomic level (by residential area)			.015*
Low	33 (49.3%)	34 (50.7%)	
Middle and high^a^	86 (33.2%)	173 (66.8%)	
Missing values	6	7	
Immigration			.006*
Native-born and established immigrants	106 (34.5%)	201 (65.5%)	
Immigrants (immigrated to Israel after 1989)	19 (59.4%)	13 (40.6%)	
Country of birth			0.741
Israeli	78 (36.1%)	138 (63.9%)	
European / American	14 (37.8%)	23 (62.2%)	
Soviet Union	13 (44.8%)	16 (55.2%)	
Asian / African	18 (32.7%)	37 (67.3%)	
Missing values	2		
Perceived Health status			0.68
Very good	49 (38.9%)	77 (61.1%)	
Good	44 (34.1%)	85 (65.9%)	
Not so good	27 (34.2%)	52 (65.8%)	
Missing values	5		

*p* < 0.05

Note: Percentages are calculated as valid % per each row (i.e., each row sums up to 100%, without Missing values)

^a^High socio-economic level was combined with middle socio-economic level because of small numbers at this level.

**Table 2:** Patient characteristics associated with the setting of getting a SO: private vs. public using logistic regression model

	Survey method		
	*n* = 339		
Predictors	OR	95% CI	P
Religiosity			
Religious	Reference group		
Secular	0.45	0.27–0.76	0.03
Immigration			
Immigrants*	Reference group		
Native-born and established immigrants	3.68	1.67–8.1	0.01

### Reasons for seeking a second opinion in the private market

One hundred twenty-seven out of 214 respondents who sought a SO from a private physician, provided reasons for seeking a SO in a private setting (Table 3). The main reason for seeking a SO from a private physician rather than from the public system was an assumption that private physicians are more professional (*n* = 58, 45.7% of 127). The other reasons were prior acquaintance with the physician or a word-of-mouth about the specific private physician (*n* = 21, 16.5%), waiting time at the health fund (*n* = 18, 14.2%), that private physicians have better attitudes (*n* = 13, 10.2%) or other reasons such as flexible hours, restrictions of the public health fund, etc. (*n* = 17, 13.4%).

**Table 3:** Main reasons for seeking a SO from a private physician rather than from the public system (*n* = 127)

Reasons for seeking a SO from a private physician	*n* = 127 (%)
Private physician is more professional than the public health fund physician	58 (45.7%)
Prior acquaintance with the physician or by receiving a recommendation about the specific physician who works in a private clinic	21 (16.5%)
Waiting time at the health fund	18 (14.2%)
Private physicians have a better attitude and are more relaxing	13 (10.2%)
Other	17 (13.4%)
Total	127 (100.0%)
Missing	87

### Perceived outcomes of the consultation

Interestingly, there were no differences in the perceived outcomes between private and public consultations. Respondents who obtained a private SO were neither more satisfied from the SO, nor felt clinical improvement after the SO, nor mentioned any difference between the diagnosis or treatment between the first and the SO, nor tended to prefer the recommendation that was given in the SO over of the first opinion, compared to those who obtained the SO in a public system (Table 4).

**Table 4:** Perceived outcomes of getting a second opinion in the private vs. the public system (n = 339 respondents)

Perceived outcomes	Public *n* = 125 (%)	Private *n* = 214 (%)	*p*-value
I preferred the SO over the first one	47 (85.5%)	112 (94.9%)	0.07^1^
There was a difference between the diagnosis or treatment between the first and the SO	54 (49.1%)	113 (59.5%)	0.08
I was satisfied with the SO	96 (82.1%)	179 (85.2%)	0.45
I felt health improvement after getting the SO	53 (51.0%)	86 (45.5%)	0.37

^1^Fisher’s Exact Test

## Discussion

Public health systems in tax-funded healthcare systems, such as Medicare and Medicaid in the US, the NHS in the UK, and the public systems in Canada and Europe, offer free or affordable access to health services. Such services strive for high quality, but operate under limitations of public budgets which limit choice and quality. In theory, private healthcare services overtake public services in those dimensions, offering allegedly higher level of expertise, more choices, better quality, shorter waiting times, and a sense of exclusivity, for people who can afford them. Yet, our findings shed a different light on this assumption. Although the main reason for seeking a SO from a private physician was the assumption that private physicians are more professional, those who sought a SO from a private physician were neither more satisfied from the SO nor felt that their symptoms have more improved nor their diagnosis or treatment were different after the SO. Hence, the underlying ‘story’ here is how patient perceptions on the superiority of the private market draw them to seek private SOs. The private sector benefits from those perceptions, by providing an answer to those patients who can afford to pay for private services. Our findings support other systematic reviews and studies negating the belief that the private sector is more efficient, accountable, or medically more effective than the public one [32, 33]. Particularly in Israel, there is no evidence that patients with supplementary programs receive better services than ones with a public insurance [8]. So, what attracted most of the respondents to seek a SO on the private market?

### What attracts patients to the private market?

First, private physicians provide access to expertise. People usually seek a SO when their problem is complex, and they would sensibly look for a renowned expert. Private insurances enable easier access to senior specialists (e.g., department heads) and provide the freedom of choosing a specific physician, which is not necessarily possible in the public sector. Second, patients may feel it is worth paying for private consultations to shorten waiting times [34]. Waiting times to specialists can be rather lengthy, and in some health funds in Israel, for example in Clalit Health Services (the largest Israeli HMO), patients can see a different specialist only after 3 months have passed from the first consultation with a specialist in a particular medical domain. Finally, patients sought SOs from private specialists due to the sense that private physicians have a better attitude to patients. In a private consultation, patients are paying for the physician’s time in a relaxed, hopefully heedful consultation, where the physician has more time to probe into the patient’s concerns. Consequently, patients can build better relationships with the specialists which result in higher levels of trust [35]. While our findings negate the assumption that the private market excels over the public one, at least in regards to SOs, it probably excels on the patient-centeredness dimension. Private practices can be more responsive in aspects of care delivery and are being more client orientated [36, 37]. Such environments can be seldom provided by public systems which are commonly hasty.

There is a bit of an irony here, because many specialists in Israel work in both public and private settings, even on the same day (usually mornings in public settings and afternoons in private settings). This may provide a partial explanation for the lack of differences in clinical judgment between the two settings. Yet the same clinician, who works in both settings, can offer a much better experience in their private practice, in terms of shorter waiting times and a more relaxed and attentive atmosphere.

### Differences in utilization of second opinions in the private vs public systems

In our final model we found that immigrants (less than 28 years in Israel) tend to seek more SOs from the public system compared to the private one. A previous study showed that immigrants use less health care services, although they enjoy the same health care coverage as non-immigrants. This utilization is probably due to economic and cultural factors [38].

Socio-economic level was not included in the final model but there is a hint about its potential influence as it was significant in the univariate analysis. Our study showed that respondents from low socio-economic levels sought more SOs from the public sector, probably due to the higher costs in the private one. These differences can be interpreted as an inequality of health services utilization and can represent a preference of the high income group to use private health services [39]. These findings are also in line with another study showing that people with a higher socio-economic status visit more specialists [7]. Preference for private physicians driven by the assumption that they are superior to the public ones may introduce inequalities in access as private SOs are paid out of pocket (and reimbursed under some conditions for those with supplementary programs). Hence, there is a structural inequality in the health system where accessing a private SO means paying higher out-of-pocket costs and it is unfair to weaker populations. Access to private SOs can have consequences in access inequality measures such as waiting times as those who cannot afford to pay privately actually have to wait a longer time.

### Practical implications

Reducing inequalities in health has been defined as a strategic goal in western countries, including Israel. Enabling access to SOs in public systems can reduce unnecessary out-of-pocket payments for private consultations (for the patient) and their reimbursement by NHIs. A second implication, though not easy to implement, is the need to inform patients about expertise and quality of private physicians where public-private mix exists. Patients as health consumers try to maximize on quality and minimize on costs. While the cost is clear in both public and private markets, quality is difficult to measure and combines medical expertise, patient-centered approach and ease of access (i.e., a shorter waiting time and flexible appointment time). In the absence of valid information on quality of public and private clinicians, patients rely on intuition, recommendations of friends and relatives, or reviews available in the media and the internet, commonly with limited value. Lack of valid information draws patients to decisions that are not always rational [40]. Our findings indicate that patients are willing to pay more for a private consult while the perceived outcomes of satisfaction are rather similar to a physician who works in the public system. On the other hand, healthcare providers in the public sector should pay more attention to what customers really need and satisfy their expectations to timeliness and kindness [41].

### Strengths and limitations

This study is unique in exploring the relation between the utilization of SO in the private system vs the public system. Previous studies did not look into the reasons for preferring a private physician over a public one for a SO. This study has several limitations. First, our definition of a SO as ‘visiting another specialist, in the same specialty, in order to get a SO on the same medical problem during the past 12 months (excluding visits to family physicians)’ does not capture all cases of SO. For example, patients may seek a SO on the same episode from specialists in different clinical domains (e.g., seeking advice about back pain from both an orthopedic surgeon and a neurologist). We chose this definition after thorough methodological considerations, to avoid misinterpretation of the question by patients. Second, as in any survey, selection and recall biases may have occurred, as well as embarrassment and social desirability that might limit the validity of the findings. For example, patients might have felt uncomfortable to disclose intimate health conditions in a telephone survey.

## Conclusions

The study analyzed the characteristics of people seeking a SO from the public system vs. the private system. We analyzed the influencing factors on patient preferences on consulting with a private physician rather than a physician who works in a public setting, and whether the private consultation indeed delivers more objective value to the patient. Our findings shed light not only on SOs, but also on the relation between the private and public health systems in countries with a public-private mix.

Particular patient groups (religious respondents and immigrants) sought more SOs from the public sector. Low socio-economic level, which was significant only in the univariate analyses, can hint about potential inequalities in access to SOs. The main reason for seeking a SO from private physicians was the assumption that they are more professional than physicians working in public settings. However, there were no differences in satisfaction from the SO and clinical judgment between the two settings. This raises the question of the added value of getting a SO on the private market.

Considering better ways to improve the public services and better mechanisms to finance SOs through the public systems can help improve access to some social groups. In parallel, keeping improving patient-physician communication is important in order to reduce unnecessary demands for SOs. Further research is recommended to validate the survey findings with objective visits data.

## Abbreviations

HMO: Health Maintenance Organization
NHI: National Health Insurance
SO: Second Opinion
